# *De novo* ß-hairpin design using a residue-basedphysicochemical property landscape

**DOI:** 10.1063/4.0001124

**Published:** 2025-10-27

**Authors:** Vardhan Satalkar, Julie Mitchell, Mathhew Torres

**Affiliations:** 1 Georgia Institute of Technology, Atlanta, Georgia, USA; 2 University of Wisconsin, Maddison, Wisconsin, USA

## Abstract

De novo peptide design is a rapidly evolving field with significant promise for applications in both biological and biomedical sectors. Traditional peptide design methods often rely on sequence homology, which is limited to evolutionarily related proteins and fails to incorporate the key physicochemical factors necessary for proper protein folding. Generative machine learning models offer a unique opportunity to create novel peptide sequences that are inspired by existing data but are not constrained by evolutionary limitations. In this study, we developed a custom generative adversarial network (GAN) designed specifically to generate peptide sequences capable of folding into β-hairpin secondary structures. This model is built on the principles of physicochemical properties, such as hydrophobicity and residue volume, and utilizes sequence data from known structures in the PDB to guide its design process. The β-GAN model demonstrated a remarkable ability to differentiate β-hairpin structures from α-helices and disordered peptides, achieving an accuracy rate of up to 96%. Furthermore, the model produced β-hairpin peptide sequences with sequence identities as low as 31% compared to the current PDB and 50% compared to non-redundant databases. These results highlight the potential of machine learning-based generative models to push the boundaries of peptide design, creating novel sequences and structures that transcend evolutionary constraints and offer exciting new possibilities in the field.

